# Patient experiences of methadone treatment changes during the first wave of COVID-19: a national community-driven survey

**DOI:** 10.1186/s12954-023-00756-3

**Published:** 2023-03-09

**Authors:** Sarah Brothers, Adam Palayew, Caty Simon, Abby Coulter, Knina Strichartz, Nick Voyles, Louise Vincent

**Affiliations:** 1 Methadone Advocacy Working Group, National Survivors Union, Greensboro, NC USA; 2 Department of Sociology, Pennsylvania State University, University Park, USA; 3 NC Survivors Union, Greensboro, NC USA; 4 Department of Epidemiology, University of Washington, Seattle, Washington USA; 5 Whose Corner Is It Anyway, Holyoke, MA USA

**Keywords:** United States, COVID-19, Methadone, Opioid use disorder, Community-driven research

## Abstract

**Background:**

During COVID-19, the Substance Abuse and Mental Health Services Administration (SAMHSA) allowed Methadone Maintenance Treatment (MMT) programs to relax in-person MMT requirements to reduce COVID-19 exposure. This study examines patient-reported changes to in-person methadone clinic attendance requirements during COVID-19.

**Methods:**

From June 7, 2020, to July 15, 2020, a convenience sample of methadone patients (*N* = 392) were recruited in collaboration with National Survivors Union (NSU) in 43 states and Washington D.C. through social media (Facebook, Reddit, Twitter, and Web site pop-ups). The community-driven research (CDR) online survey collected information on how patient take-home methadone dosing and in-person drug testing, counseling, and clinic visit frequency changed prior to COVID-19 (before March 2020) to during COVID-19 (June and July 2020).

**Results:**

During the study time period, the percentage of respondents receiving at least 14 days of take-home doses increased from 22 to 53%, while the percentage receiving one or no take-home doses decreased from 22.4% before COVID-19 to 10.2% during COVID-19. In-person counseling attendance decreased from 82.9% to 19.4%. While only 3.3% of respondents accessed counseling through telehealth before COVID-19, this percentage increased to 61.7% during COVID-19. Many respondents (41.3%) reported visiting their clinics in person once a week or more during COVID-19.

**Conclusions:**

During the first wave of COVID-19, methadone patients report decreased in-person clinic attendance and increased take-home doses and use of telehealth for counseling services. However, respondents reported considerable variations, and many were still required to make frequent in-person clinic visits, which put patients at risk of COVID-19 exposure. Relaxations of MMT in-person requirements during COVID-19 should be consistently implemented and made permanent, and patient experiences of these changes should be explored further.

## Introduction

During the COVID-19 pandemic, over 26 million people in the world who live with opioid use disorder (OUD) [16] are particularly vulnerable. They are at increased risk for negative health consequences from COVID-19 due to high rates of comorbidities such as cardiovascular and respiratory diseases [43, 59] and other negative consequences of COVID-19 including negative respiratory effects, hospitalization, and mortality from COVID-19 [4]. This population is also at a high risk of overdose, suicide, housing instability, food insecurity, and unemployment, and COVID-19 has exacerbated these issues [2, 15, 43, 59].

In the United States, 1.6 million people were living with OUD in 2019 [52]. OUD rates are increasing, as are HCV infections and fatal drug overdoses [1, 17, 27]. Clinical evidence shows that methadone is the most empirically proven treatment available in the United States for OUD [13]. Methadone maintenance treatment (MMT) improves mental and physical health, eliminates opioid withdrawal symptoms, and has few toxic side effects [37, 49]. It decreases overdose risk, HIV and HCV infections, illegal substance use, and overall morbidity and mortality [49, 61]. However, in 2019, only 408,550 people received MMT in the United States [53].

Even though MMT is a safe and effective treatment for OUD, it is heavily regulated in the United States. Unlike buprenorphine maintenance treatment (BMT), which can be prescribed by primary care providers and dispensed from pharmacies, only certified opioid treatment programs (OTPs) can dispense methadone [24, 31]. Doses are typically given to patients in-person under daily direct supervision, with regular drug testing and counseling sessions required [70]. Thus, MMT places a heavy time burden on patients, decreasing treatment retention and quality of life and making employment and rehabilitation more difficult [57, 62, 71]. Additionally, MMT can be costly, which decreases treatment retention [7, 35].

In response to COVID-19, in March 2020, the Substance Abuse and Mental Health Services Administration (SAMHSA) relaxed MMT requirements to decrease COVID-19 exposure risk by reducing in-person visits for medication, drug testing, and counseling [51]. This letter aimed to address concerns that social distancing was difficult, if not impossible, to maintain given existing MMT practices, including in-person visits for medication, drug testing, and counseling. The guidance allowed 28 days of unsupervised take-home dosing for stable patients, and up to 14 days for those considered less stable. U.S. federal regulations recommend OTPs use the following criteria to determine patient stability: length of treatment, take-home dose benefits outweigh risks, treatment plan adherence, no recent positive toxicology tests or substance-use-related behaviors, no behavioral issues, stable housing and relationships, no recent diversion history, and safe storage of MMT [54]. OTPs defined patient stability with considerable variation during the first wave of COVID-19 [8, 26, 63]

On April 21, 2020, it was further recommended that OTPs increase telehealth use to further reduce potential COVID-19 exposure [2, 28].

Recent studies find that during COVID-19, MMT clinic visits and toxicology screens decreased, and take-home doses and telehealth use increased with no increase in fatal MMT-related overdose or severity of methadone poisoning exposure and little reported diversion [8, 21, 23, 25, 33, 40, 44, 69]. Increased take-home dosing during COVID-19 was associated with decreased illicit drug use and increased retention [19, 29]. Most studies have focused on clinician perspectives [30, 39, 66]. Studies on methadone patient experiences of treatment changes during COVID-19 are limited to states, counties, and individual OTPs or examine time periods after July 2020 [45, 64, 65, 68]. Little is known about MMT patient experiences of in-person treatment requirement changes across the United States during the first wave of COVID-19. In this article, we use a community-driven research (CDR) approach to examine MMT patient-reported experiences of four issues that put them at a high risk of COVID-19 exposure during the first months of COVID-19: take-home dosing limits, in-person clinic visits, drug testing, and on-site counseling requirements.

## Methods

Consistent with the community-driven research (CDR) approach, this project was designed and led by people directly impacted by methadone treatment policies [47, 58]. The first author, in collaboration with National Survivors Union (NSU), the national union for people who use drugs in the United States, used a CDR [47] approach to the study. CDR is particularly useful for research with marginalized populations who often have negative experiences with researchers [11]. In the CDR model, members of the impacted community are considered fundamental drivers of all aspects of the research, from initiating and developing the research questions to data interpretation, analysis, and dissemination phases of the project [9]. Our use of the CDR model emphasizes leadership capacity development for community members with living experience [58]. Since the early stages of the project were unfunded, in lieu of compensating directly impacted collaborators, the first author provided NSU members with training and contributed to NSU activities unrelated to the research.

Data for these analyses come from a national online survey NSU conducted to discover if and how MMT in-person requirements were relaxed throughout the country. The cross-sectional survey, written in English, contained 28 questions including two write-in response questions (Appendix). NSU members designed the survey questions based on their MMT experiences during COVID-19 or the experiences of methadone patients in their community. Next, an academic researcher member phrased the questions, which six NSU methadone workgroup members evaluated over four two-hour sessions and further refined. Two members tested the survey prior to distribution for inclusive and accessible language, survey length, and potentially stigmatizing or traumatizing questions. As a result of testing, the survey was shortened, phrasing and word choice changed for several questions, and write-in questions were added.

The survey was conducted from June 7, 2020, to July 15, 2020. NSU members used targeted sampling methods [46] to recruit a convenience sample of methadone patients through drug use and methadone patient social media groups (Facebook, Reddit, Web site pop-ups, and Twitter). No respondents were compensated for participation because the CDR project was unfunded. All questions were optional. The anonymous survey was short (approximately 7 min) in respect of participants’ time.

In total, 455 people participated in the survey. The study inclusion criteria were self-reported current methadone treatment in the United States. Responses were checked to ensure respondents submitted only one response. We omitted respondents who did not complete the survey or reported that they were not MMT patients in the United States. The final analytic sample comprised 392 participants from 219 cities (1 to 9 participants per city) in 43 states and Washington D.C.

### Data collection

The survey collected sociodemographic information including age-group, gender identity, race/ethnicity, health insurance type, monthly out-of-pocket payments for methadone, and participant MMT clinic state and city. It also collected self-reported information on take-home dose quantities, in-person clinic visits, toxicological screens, and counseling attendance.

Participant take-home dose quantities were measured categorically as: no take-home doses, 1 per week, 2 per week, 3 per week, 4 per week, 5 per week, 6 or 7 (one week), 13 or 14 (two weeks), and 27 or 28 (one month). Overall clinic attendance was measured categorically as every day, 6 times a week, 5 times a week, 4 times a week, 3 times a week, 2 times a week, once a week, twice a month, or once a month. Counseling attendance was also measured categorically as: daily, 3 times a week, 2 times a week, weekly, 6 times a month, 3 times a month, 2 times a month, once a month, once every 3 months, and none. Method of counseling attendance was also measured categorically as: not required to attend counseling services, in person, through telehealth, by telephone, and none. Information regarding how often toxicological screens were required was measured as, “never,” “1–2 times a month,” “2–5 times a month,” “5–7 times a month,” “7–10 times a month,” “10–15 times a month,” “20 +, ” and “other” as a write-in response. Items where participants indicated they did not know or if they left the question blank, that response was considered missing. Categories were established by consulting with community members and with considerations toward the balance of sample size between groups. The survey included two write-in response questions: “What has your clinic done to maintain 6-foot social distancing between people?” and “Is there anything else you would like to tell us about your clinic's practices during COVID-19?”.

### Statistical analysis

Descriptive statistics were calculated for changes in required clinic visits and drug screening frequency, number of take-home doses, and counseling during COVID-19. States were grouped into four regions following the United States Census Bureau definition [67]. Insurance was grouped into three categories: private, government, and none. The private insurance category includes company healthcare plans, insurance purchased through the Affordable Care Act Health Insurance Marketplace (“Obamacare”), and self-funded insurance. The government-funded insurance category includes Medicaid, Medicare, Tricare, MassHealth, New Jersey family care, and state and county grants. People who identified two race/ethnicity categories were counted according to their minority category (for example, someone who identified as Black and white was counted as Black).

We used logistic regression models to examine factors associated with two outcomes related to in-person clinic attendance. The first outcome was whether someone received increased methadone take-home doses. Participants who did not report increased take-home doses during COVID-19 were coded as 0. Participants who reported any increase in take-home doses during COVID-19 were coded as 1. The second outcome was whether someone reported decreased in-person counseling. Participants who reported switching from in-person counseling prior to COVID-19 to telehealth or to no counseling during COVID-19 were coded as 1. Anyone who reported that they maintained in-person counseling or switched to in-person counseling during COVID-19 was coded as 0.

We looked at the associations of these two outcomes with the covariates of gender, age, region, methadone cost, and time in MMT in univariate logistic regression outcomes. We fitted a second set of logistic regressions for the same outcomes and covariates, and we accounted for time on MMT by including it as a covariate. Those who had missing data were excluded from the regression analyses.

## Results

The total sample included 392 participants who reported currently receiving MMT in the United States (Table 1). A plurality came from the United States South (42.1%), with the smallest number coming from the West (8.2%) (Table 1). The majority (76.0%) identified as female, 85.9% identified as white, and 67.8% were below the age of 40. MMT treatment duration was less than 5 years for 46.4% of participants. Almost one-third (31.1%, *n* = 122) of participants paid $100 or more for MMT monthly (Table 1).

**Table 1:** Demographic characteristics of methadone patients, United States 2020 (*N* = 392)

Region*	
West	8.2% (32)
South	42.1% (165)
Midwest	21.2% (83)
Northeast	26% (102)
Declined to state	2.6% (10)
*Gender Identity*	
Male	18.4% (72)
Female	76.0% (298)
Trans, nonbinary, genderqueer, other	5.4% (21)
Declined to state	0.3% (1)
*Race/Ethnicity*	
White	85.9% (336)
Black	2.0% (8)
Hispanic	4.6% (18)
Native American/Inuit	3.3% (13)
Asian/Asian American	1.5% (6)
Declined to state	2.6% (10)
*Age*	
< 30	19.2% (75)
30–39	48.8% (191)
40–49	22.5% (88)
50 +	9.5% (37)
Declined to state	0.3% (1)
*Time in Methadone Treatment*	
< = 1 year	9.7% (38)
1–4 years	36.7% (144)
5–10 years	25.8% (101)
10 + years	23.7% (93)
Declined to state	4.1% (16)
*Travel Time*	
< 20 min	48% (188)
20–45 min	30.9% (121)
45 + minutes	18.9% (74)
Declined to state	2.3% (9)
*Transportation Type*	
Alone (driving, walking, biking)	66.5% (260)
Group (Carpool, public transport)	29.4% (115)
Declined to state	4.1% (16)
*Monthly Methadone Cost*	
$0-$19	62.8% (246)
$20–99	3.3% (13)
$100–199	6.9% (27)
$200 +	24.2% (95)
Declined to state	2.8% (11)
*Health Insurance Type*	
Government-funded	58.9% (231)
No Insurance	13.0% (51)
Private (company health care plan, Obama care)	26.8% (105)
Declined to state	1.3% (5)

^*^States not represented in sample: HI, ID, MT, NE, NV, ND, WY

Half of the respondents reported increased take-home doses during COVID-19 (50.4% (185/367), data not shown). Patients receiving 28-day take-home doses increased 2.5-fold (*n* = 27 before, *n* = 95 during) (Table 2). The number of patients receiving one or no take-home doses decreased from 22.4 to 10.2%.

**Table 2:** Changes in methadone patient in-person treatment

Changes in Patient Treatment	Before COVID-19	During COVID-19	*P*-value
*Take-home doses*			< 0.001
28-day take-home doses	6.9% (27)	24.2% (95)	
14 to 21-day take-home doses	15.1% (59)	28.8% (113)	
2 to 7-day take-home doses	50.5% (198)	30.9% (121)	
None or 1 take-home doses	22.4% (88)	10.2% (40)	
Declined to state	5.1% (20)	5.9% (23)	
*Clinic attendance*			< 0.001
Never	0%	0.8% (3)	
Two or less times a month	21.2% (83)	53.3% (209)	
Once a week	26% (102)	17.9% (70)	
2–6 times a week	19.9% (78)	9.9% (39)	
Everyday	27.8% (109)	13.5% (53)	
Declined to state	5.1% (20)	4.6% (18)	
*Drug testing*			< 0.001
Less than once a month	0.3% (1)	2.8% (11)	
1–2 times a month	76.5% (300)	71.4% (280)	
2 or more times a month	13.3% (52)	10.5% (41)	
Random	3.1% (12)	2.6% (10)	
Never	1.3% (5)	9.2% (36)	
*Counseling*			< 0.001
In person	82.9% (325)	19.4% (76)	
Telehealth	3.3% (13)	61.7% (242)	
Not required	8.2% (32)	14% (55)	
Decline to state	5.6% (22)	4.8% (19)	

**Fig. 1:**
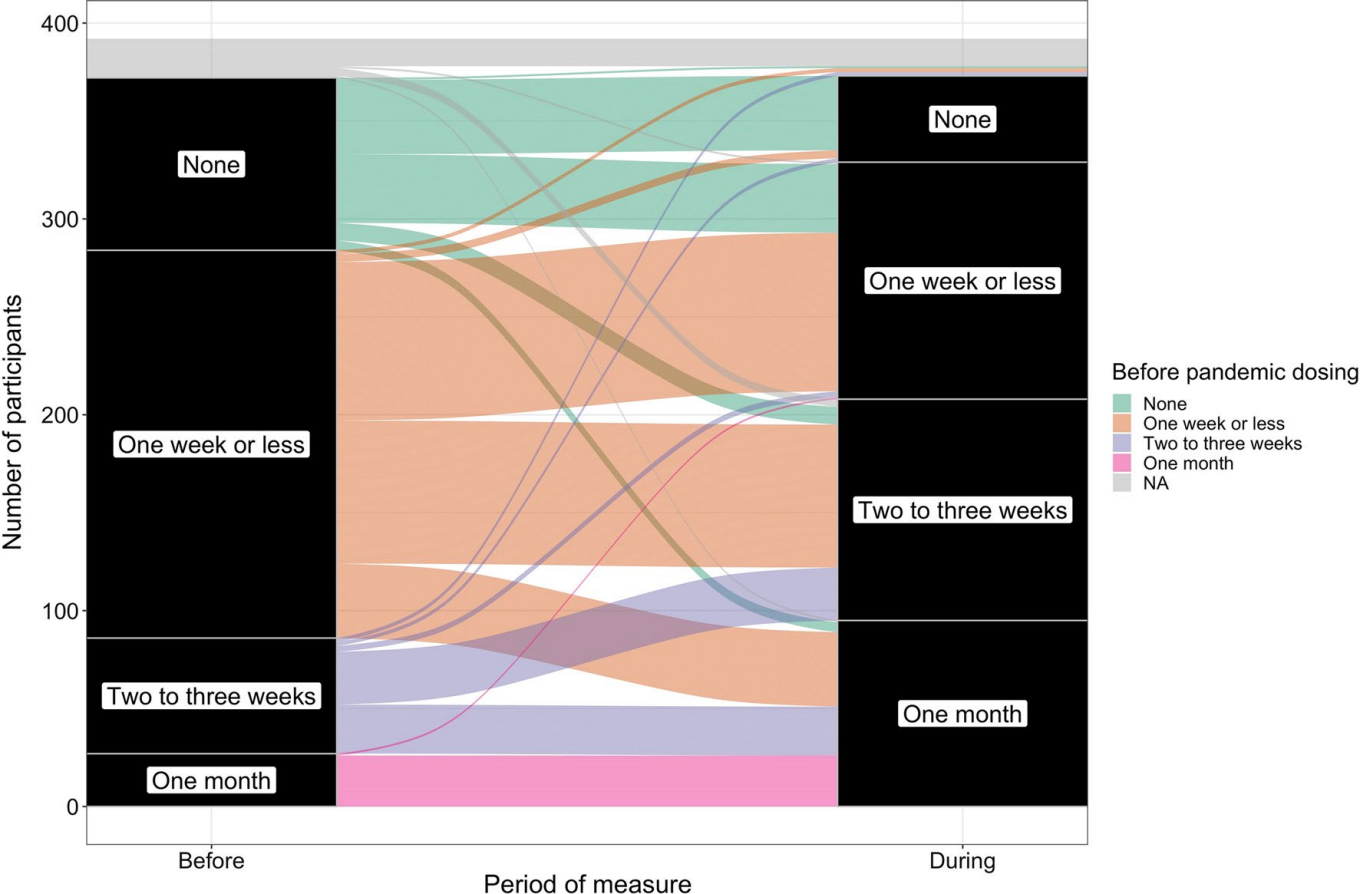
Alluvial plot of changes in clinic visit frequency

MMT clinic attendance decreased during COVID-19. Before COVID-19, 73.7% of respondents reported weekly or more frequent attendance; during COVID-19 this percentage dropped to 41.3%. The percentage of patients attending their clinics two or less times a month increased from 21.2 to 53.3%. In total, 53.0% of respondents attended their clinic less frequently during COVID-19 than before COVID-19 (data not shown). Although clinic attendance decreased overall, some patients experienced no decrease, and some experienced an increase in clinic attendance (Fig. 1).

In-person counseling attendance decreased from 82.9% before COVID-19 to 19.4% during COVID-19. Correspondingly, while only 3.3% of respondents accessed counseling through telehealth before the pandemic, this percentage increased to 61.7% during COVID-19 (Table 2). The percentage of patients who said they were not required to attend counseling services or that services were not available increased from 8.2% before COVID-19 to 14.0% during COVID-19.

We received a write-in response from 23 respondents in 12 states (Kentucky, Indiana, Illinois, Maryland, Massachusetts, Oklahoma, Maine, Florida, Pennsylvania, Michigan, North Carolina, Ohio) that their clinics had returned to pre-COVID-19 practices and eliminated increased take-home doses.

### Regression analysis of factors associated with take-home doses and in-person counseling

We found that there were no associations for increased take-home doses by age, gender, cost of treatment, or length of time in MMT (Table 3). For the counseling outcomes, those paying more than $100 out of pocket for treatment were more likely to be required to attend in-person counseling sessions than those paying less than $20 out of pocket. These associations changed minimally when controlling for the length of time in treatment (Table 3).

**Table 3:** Regression outcomes of associations

	Outcome: increase in take-home doses		Outcome: decrease in in-person counseling requirement	
	Univariate association OR [95%CI]	Controlled for methadone treatment length OR [95%CI]	Univariate association OR [95%CI]	Controlled for methadone treatment length OR [95%CI]
*Age*				
30–39 vs < 30	0.97 [0.56, 1.67]	0.84 [0.47, 1.50]	1.62 [0.85, 3.04]	1.52 [0.78, 2.93]
40–49 vs < 30	0.74 [0.39, 1.40]	0.63 [0.31, 1.24]	0.89 [0.44, 1.80]	0.81 [0.38, 1.70]
50 + vs < 30	1.58 [0.68, 3.77]	1.28 [0.51, 3.31]	0.82 [0.33, 2.10]	0.79 [0.29, 2.21]
*Gender*				
Female vs Male	1.35 [0.79, 2.35]	1.35 [0.78, 2.35]	0.82 [0.41, 1.53]	0.82 [0.41, 1.55]
Other vs Male	1.01 [0.36, 2.76]	0.99 [0.35, 2.74]	0.54 [0.18, 1.65]	0.53 [0.18, 1.62]
*Cost*				
20 – 99$ vs < 20$	0.57 [0.17, 1.75]	0.50 [0.15, 1.58]	1.34 [0.34, 8.84]	1.25 [0.32, 8.30]
100 – 199$ vs < 20$	0.73 [0.32, 1.62]	0.60 [0.25, 1.37]	0.23 [0.10, 0.52]	0.20 [0.08, 0.46]
> 200$ vs < 20$	0.85 [0.52, 1.39]	0.78 [0.47, 1.28]	0.55 [0.32, 0.95]	0.51 [0.29, 0.89]
*Region*				
Northeast vs Midwest	1.31 [0.72, 2.37]	1.31 [0.72, 2.39]	2.61 [1.22, 5.84]	2.54 [1.18, 5.70]
South vs Midwest	1.24 [0.72, 2.14]	1.27 [0.74, 2.19]	0.92 [0.50, 1.65]	0.94 [0.51, 1.70]
West vs Midwest	0.61 [0.25, 1.41]	0.64 [0.26, 1.49]	0.71 [0.29, 1.80]	0.71 [0.29 1.83]
*Length on methadone treatment*				
1–5 years vs < 1 year	1.51 [0.73, 3.19]	–	1.07 [0.45, 2.37]	–
5–10 years vs < 1 year	1.57 [0.74, 3.41]	–	1.30 [0.53, 3.04]	–
> 10 years vs < 1 year	2.01 [0.93, 4.41]	–	1.16 [0.47, 2.73]	–

## Discussion

This national survey utilizes a CDR approach to describe methadone patient experiences of MMT in-person requirement changes during the first wave of COVID-19 in the United States. During COVID-19, patients reported decreased in-person clinic attendance and increased take-home doses and telehealth use for counseling, similar to the findings of a multi-state survey on substance use disorder treatment experiences during COVID-19 [55]. These changes are in line with recommendations that telehealth and increased take-home doses should be implemented to decrease COVID-19 infection risk [2, 10] for patients and staff.

We found many respondents were still required to visit their clinic in person at least once a week, and many received less than two weeks of take-home doses. Some respondents reported increased in-person clinic visit requirements during COVID-19. This may be because their OTP now defined them as less stable. Some OTPs may have increased in-person attendance requirements because of liability concerns about patients’ management of expanded take-homes or lost revenues due to reduced in-person dosing [64, 65, 72]. Since studies have found that unsupervised take-home dosing does not differ from supervised in-person dosing in treatment retention, illicit opioid use, diversion, or patient deaths, direct dosing supervision may add little if any additional protection [56] and it significantly increased patients’ risk of exposure to COVID-19.

Almost 20% of the respondents were required to attend counseling sessions in person during COVID-19. However, the number of methadone patients who said they were not required to attend counseling services or that services were not available increased from 8.2 to 14% during COVID-19. Although some studies find that mandatory counseling may negatively affect patient attitudes toward treatment (WHO, 2004) other studies find that counseling may increase treatment retention and decrease opioid use and HIV risk [18, 34]. Switching counseling to telehealth would enable clinics to provide patients with its potential benefits while maintaining social distancing.

Almost one-third of respondents paid over $100 a month out of pocket for methadone. Paying high amounts for methadone treatment may decrease treatment retention, as studies have found [7, 35], especially for those experiencing financial insecurity during COVID-19. We also found that these respondents were more likely to attend in-person counseling relative to those paying less. This may be because in most states OTPs receive larger reimbursements for multiple in-person patient visits weekly [30, 63, 72].

We also found that many respondents traveled long distances via shared transport for MMT, increasing COVID-19 exposure risk. Travel time to clinics has long been shown to cause hardship for daily methadone patients [41] and decreased treatment retention [5], issues that may have worsened during COVID-19.

Patients experienced inconsistent implementation of MMT relaxations during the first months of COVID-19, as a contemporary North Carolina study also found [21]. Studies later in 2020 on OTP staff and management perspectives also showed relaxations were unevenly implemented [38, 42]. MMT relaxations varied considerably by State. Research conducted in Arizona, a state underrepresented in this study, found that most patients did not receive reduced in-person requirements or 14- or 28-day take-home doses [45]. Notably, some respondents in our study said their clinics had revoked increased take-home doses during the time of this survey (June/July 2020), just a few months after SAMHSA released its initial guidance to increase take-home dosing access. Treatment requirements may have made it difficult for people to enter or remain in treatment while protecting their health and reducing the spread of COVID-19. Stronger state and federal guidance is needed for relaxing MMT requirements, including reducing in-person group and individual counseling requirements, transitioning to telehealth for services, and increasing take-home doses consistently.

During COVID-19, it is critical to remember that we are still in the midst of an overdose crisis, and overdose rates are increasing across the USA [14, 60]. One treatment that has consistently proven to greatly reduce the risk of overdose is methadone. While the new federal SAMHSA guidelines are a step forward, we have not yet seen consistent relaxation of barriers to treatment for methadone patients across the United States. Researchers have long called for expanding access to methadone treatment, reducing supervised in-person dosing, and decreasing regulations and the burden of compliance and attendance [50]. Given that methadone-related fatal overdose rates have not increased since the MMT relaxations were implemented [8, 32], the COVID-19 relaxations in MMT requirements should be further relaxed and made permanent, as experts have also recommended [14]. National Institute on Drug Abuse Director Nora Volkow recently made a statement supporting office-based prescription and pharmacy dispensing of methadone [20]. Allowing methadone treatment in community pharmacies would also help expand access to methadone treatment, reduce travel times, and help address the opioid epidemic and issues arising from COVID-19 [12]. Given COVID-19 and the overdose crisis, increasing methadone access, and reducing barriers to treatment is all the more urgent.

### Limitations

Our study has limitations. Since this CDR study was initially unfunded, full participation in the research process was less accessible to NSU members who could not afford to donate their time. Our current projects prioritize compensation for all participants. The questionnaire relied on self-reported information, which raises reliability and validity questions about the findings; however, we have no reason to believe that participants were not truthful. Furthermore, this cross-sectional study represents patient experiences of MMT changes during the survey time period. Program changes continue to evolve over time. Additionally, this convenience sample of methadone patients recruited through social media who self-selected into the survey and were not compensated for participation may not be representative. Although our sample is not clustered by state or city (no more than 9 people participated from any city), the sample may not be representative by race/ethnicity, gender identity, time in treatment, or other variables. We did not disseminate a Spanish language survey, which may have led to portions of the United States being underrepresented. Moreover, we do not have enough data on non-white participants to describe any racial disparities, which is another limitation of the sample. Also, many factors likely to influence take-home regimens, including stability measures, were not collected. Finally, MMT restriction relaxations may depend on clinic protocols rather than patient characteristics.

## Conclusion

Methadone patients report considerable variation in how relaxations in methadone treatment have been implemented during COVID-19. Although some patients received increased take-home doses and decreased in-person clinic attendance requirements, many reported frequent in-person clinic visits. Further research should explore the reasons for the variation in MMT relaxation implementation during COVID-19. OTPs should be encouraged to permanently and consistently reduce MMT barriers, particularly in-person attendance requirements.

## Data Availability

The data and code to reproduce this analysis can be found at https://osf.io/f6zw2/.

## Appendix

Methadone patient online survey

**Table:**

1. How old are you?	18–25
	25–30
	30–35
	35–40
	40–45
	45–50
	50 +
2. What is your current gender identity?	Cis Man
	Cis Woman
	Agender
	Nonbinary
	Trans Man
	Trans Woman
	Other (*write-in response*)
3. Which of the following best describes your race? Please check all that apply	Native American
	African American
	Asian/Asian American
	Caucasian
	Hispanic
	Pacific Islander
	Prefer not to answer
	Other (*write-in response*)
4. What best describes your health insurance?	Medicaid/Medicare
	Government Healthcare (Obama Care)
	Self-Pay Private Insurance
	Company Healthcare Plan
	I don't have health insurance
	Other (*write-in response*)
5. How much do you pay out of pocket per month for methadone?	$0-$20
	$20-$40
	$60-$80
	$100-$120
	$140-$160
	$160-$180
	$180-$200
6. What state is your clinic located in?	
7. What city is your clinic located in?	
8. How many miles away is the clinic from your home?	
9. How many minutes is the travel time each way?	0–20 min
	20–30 min
	30–45 min
	45–60 min
	60–75 min
	75–90 min
	over 90 min
10. How do you get to your clinic? Please check all which apply	Public transportation bus
	Public transportation train
	Walk
	Bike
	Carpooling with others
	Driving by self
11. How long have you been prescribed methadone?	1–3 months
	3–6 months
	6–9 months
	9 months-1 year
	1–3 years
	3–5 years
	5–7 years
	7–10 years
	10 + years
12. How often did you go to the clinic before the pandemic?	Every day
	3 times a week
	2 times a week
	Once a week
	Twice a month
	Once a month
	Other (*write-in response*)
13. How often do you go to the clinic NOW?	Every day
	3 times a week
	2 times a week
	Once a week
	Twice a month
	Once a month
	Other (*write-in response*)
14. How many take-home doses did you receive BEFORE the pandemic?	I did not receive take-homes
	2 weekends
	3 on Thursdays
	7 (one week)
	14 (two weeks)
	28 (one month)
	Other (*write-in response*)
15. How many take-home doses do you receive NOW?	I do not receive take-homes
	3 weekends
	4 on Thursdays
	8 (one week)
	15 (two weeks)
	29 (one month)
	Other (*write-in response*)
16. Were you required to attend any of the following at your clinic BEFORE the pandemic? Please check all which apply	Individual counseling
	Group counseling
	Family counseling
	Court ordered drug counseling
	I was not required to attend any type of counseling
	Other (*write-in response*)
17. How did you attend required counseling services BEFORE the pandemic?	I wasn't required to attend counseling services
	In person
	Through Telehealth
	Other (*write-in response*)
18. How often during a month did you attend these counseling services BEFORE the pandemic?	
19. How do you attend counseling services NOW?	I wasn't required to attend counseling services
	In person
	Through Telehealth
	Other (*write-in response*)
20. How often during the month do you attend counseling services NOW?	
21. Were you required by your clinic to participate in mandatory drug screens BEFORE the pandemic?	Yes
	No
22. How often were you required to take a drug screen BEFORE the pandemic?	Never
	1–2 times a month
	2–5 times a month
	5–7 times a month
	7–10 times a month
	10–15 times a month
	20 +
	Other (*write-in response*)
23. How often are you required to take a drug screen NOW?	Never
	1–2 times a month
	2–5 times a month
	5–7 times a month
	7–10 times a month
	10–15 times a month
	20 +
	Other (*write-in response*)
24. Is your clinic trying to maintain the social distancing at a space of 6 feet?	Yes
	No
	Sometimes
	Other (*write-in response*)
25. What has your clinic done to maintain 6-foot social distancing between people?	
26. Is there anything else you would like to tell us about your clinics’ practices during COVID-19?	
